# Single dose of gentamicin in combination with metronidazole versus multiple doses for prevention of post-caesarean infection at Bugando Medical Centre in Mwanza, Tanzania: a randomized, equivalence, controlled trial

**DOI:** 10.1186/1471-2393-13-123

**Published:** 2013-05-31

**Authors:** Fadhili M Lyimo, Anthony N Massinde, Benson R Kidenya, Eveline T Konje, Stephen E Mshana

**Affiliations:** 1 Department of Obstetrics and Gynaecology, Catholic University of Health Sciences and Allied Science, Box 1464, Mwanza, Tanzania; 2 Bugando Medical Centre, Box 1370, Mwanza, Tanzania; 3 Department of Biochemistry, Catholic University of Health Sciences and Allied Sciences, Mwanza, Tanzania; 4 Department of Epidemiology and Biostatistics, Catholic University of Health Sciences and Allied Sciences, Mwanza, Tanzania; 5 Department of Microbiology/Immunology, Catholic University of Health Sciences and Allied Sciences, Mwanza, Tanzania

**Keywords:** Post-caesarean infection, Metronidazole, Gentamicin, Mwanza, Tanzania

## Abstract

**Background:**

Caesarean section(C/S) has been found to increase rates of maternal infectious morbidities five times more than vaginal delivery. The provision of intravenous prophylactic antibiotics 30 to 60 minutes prior to C/S has been found to substantially reduce post-caesarean infection. At Bugando Medical Centre, there is no consistent protocol for the administration of antibiotic prophylaxis to patients who are undergoing emergency C/S. Providing repeated dosages of antibiotic prophylaxis after C/S is the common practice. This study aimed to determine the comparative efficacy of a single dose of gentamicin in combination with metronidazole versus multiple doses for prevention of post-caesarean infection.

**Methods:**

From October 2011 to May 2012, a randomized, equivalence, non-blinding clinical trial was conducted at Bugando Medical Centre in Mwanza, Tanzania. A total of 500 eligible participants were enrolled in the study and were randomly allocated into two study arms -- “A” and “B”. Participants in “A” received a single dose of gentamicin in combination with metronidazole 30 to 60 minutes prior to the operation, and participants in “B” received the same drugs prior to the operation but continued with for 24 hours. Both groups had 30 days of follow-up and were assessed for signs and symptoms of surgical-site infection as the primary outcome. The equivalence margin was set at 5%. The two-tailed equivalence was analyzed based on intention- to-treat analysis.

**Results:**

The randomization was proper, as the distribution of various demographic and other baseline characteristics had a p-value of > 0.05. All 500 participants were included in our analysis; of these, no participants were lost to follow-up. Surgical-site infection occurred in 12 out of the 250 (4.8%) receiving single dose compared to 16 out of the 250 (6.4%) receiving multiple doses. There is an absolute proportion difference of 1.6% (95% Confidence interval: -2.4 – 5.6%) which lies outside the pre-specified 5% equivalence margin.

**Conclusion:**

We recommend the administration of pre-operative single dose antibiotic prophylaxis for emergency caesarean as this intervention proved to be not equivalent to multiple doses antibiotic prophylaxis in reducing surgical site infection. Single dose therapy also reduces staff workload along with medication costs.

**Trial registration:**

Current Controlled Trials ISRCTN44462542

## Background

Postpartum infection remains to be among the top five causes of pregnancy-related maternal mortality and morbidity worldwide [1-3]. Women who undergo caesarean section have a 5 to 20 fold greater risk of postpartum infection than women having a vaginal delivery [4-7]. The incidence of post-cesarean infection varies widely worldwide – ranging from 2.5% to 20.5% [8-12].

Selection of antibiotics for prophylaxis follows the principle that the selected antibiotic regimen should have activity against microbial agents commonly involved in surgical-site contamination and actual infection. A combination of clindamycin and aminoglycosides has been recommended as the treatment of choice in post-caesarean infection, since it covers most of the pathogenic bacteria commonly involved in post-caesarean infection. Alternatively, a combination of metronidazole with aminoglycosides has been found to be effective [13-15]. Apart from being a drug of choice in treating gram-negative bacteria, methicillin sensitive *Staphylococcus aureus* responds to gentamicin [15,16].

A number of well-designed studies have documented the efficacy of prophylactic antibiotics in reducing the rates of post-partum infection among patients who have undergone caesarian section [8-12]. Administration of antibiotic prophylaxis within an hour prior to skin incision is more effective in reducing post-caesarean infectious morbidity when compared to administration of the same drugs after cord clamping and has no effect on neonatal infection [17-21]. In a systematic review, Owens et al. reported that provision of antibiotic prophylaxis for caesarean section before skin incision compared with after umbilical cord clamping is associated with a 40% decrease in postpartum endometritis and a 30% decrease in wound infection [22]. Provision of a single dose of antibiotics preoperatively has been found to be as effective as multiple doses in prevention of postpartum infection [23-25].

At Bugando Medical Centre (BMC), the rate of caesarian is estimated to be 20% [26]. All patients who deliver by caesarian section receive antibiotic prophylaxis. However, there is no consistent protocol for administration of antibiotic prophylaxis for patients designated for caesarean section. The common practice is to provide repeated doses of prophylactic antibiotics for at least 24 hours after caesarian section and often on individual clinicians’ practice and preference. Gentamicin and metronidazole are among the antibiotics that are used as prophylaxis for infection during caesarean delivery.

This study aimed to determine the equivalence of intravenous single dose of gentamicin (3 mg/kg) plus metronidazole (500 mg) given 30 to 60 minutes before incision and multiple doses of gentamicin (3 mg/kg) plus metronidazole (500 mg) given 30 to 60 minutes before incision then followed by gentamicin (3 mg/kg) once a day and metronidazole (500 mg) every 8 hours for 24 hours postoperatively.

## Methods

### Participants

This was an interventional, open label, two-armed, randomized, single-centre, equivalence trial conducted at BMC -- a zonal consultant and teaching hospital situated in Mwanza City, Tanzania. In this hospital, 7149 pregnant women are admitted yearly. There are 6868 deliveries per annum, and 1398 (20.3%) pregnant women deliver by caesarean section as per 2010 statistics [26].

The target population was all pregnant women who were admitted at BMC labor ward that needed or had indication for emergency caesarean section, between the dates of October 2011 and May 2012. All pregnant women who were required to undergo emergency caesarean section (under spinal anaesthesia) and had also consented for the study were eligible for inclusion, but the study excluded all pregnant women with fever (temperature of 38°C and above,) prolonged obstructed labor, and prolonged and premature rupture of membranes (rupture of membrane more than twelve hours). Pregnant women presenting with features of chorioamnionitis (i.e. foul smelling lochia, uterine tenderness associated with fever) allergies to the antibiotics used in the study, or those who had used antibiotics in the 24 hours preceding the operation or unconscious patients who could not provide consent were excluded from the study.

### Sample size estimation

Sample size was estimated according to our previously reported protocol [27]. Briefly, we used the formula for proportion difference for equivalence trial with hypothesized difference of 5% (i.e. within this difference, the two regimes are assumed to be clinically equivalent, alpha = 0.05 and beta = 0.20) [28]. By considering 10% of participants non-response or lost to follow up, the required minimum sample size was 490 (i.e. 245 participants per group). However we managed to recruit 500 participants -- 250 on each study arm.

### Randomization

Simple randomization was used to allocate study participants. A total of 500 opaque envelopes of the same size were prepared for this study; 250 envelopes contained papers marked “study arm A,” and the remaining envelopes contained papers marked “study arm B.” Before envelopes were picked, all envelopes were thoroughly mixed together in a box to allow equal chance of being selected. Each study participant selected one sealed envelope and then gave it to the research assistant to open. Antibiotic prophylaxis was prescribed according to the study arm.

### Interventions

Intervention started after allocating eligible participants into two study arms: A and B. Study arm A included those who received a single intravenous single dose of gentamicin (3 mg/Kg) plus metronidazole (500 mg) 30 to 60 minutes before operation, and study arm B included those who received multiple doses of gentamicin (3 mg/Kg) plus metronidazole (500 mg) 30 to 60 minutes before operation and metronidazole (500 mg every) 8 hours for 24 hours postoperatively.

### Specific objectives and hypothesis

The specific objective was to determine the equivalence between intravenous single dose gentamicin (3 mg/Kg) plus metronidazole (500 mg) 30 to 60 minutes before operation and multiple doses of gentamicin (3 mg/Kg) plus metronidazole (500 mg) 30 to 60 minutes before operation and metronidazole (500 mg) every 8 hours for 24 hours postoperatively.

### Null hypothesis

The treatment difference on the proportion of post-caesarian section infection in the two arms should be more than ± 5%.

### Alternative hypothesis

The treatment difference on the proportion of post-caesarian section infection in the two arms should be less than or equal to ± 5%.

### Outcomes

#### Primary outcome measure

Post-caesarean, surgical-site infection was our primary outcome. The assessment for any evidence of surgical-site infection was done 72 hours after caesarian section, as well as on follow-up days (day 7 and day 30 post-caesarean section). Two clinicians in the ward who were not involved in the study performed diagnosis of surgical-site infection. The presence of fever (febrile morbidity) signs and symptoms of abdominal wound infection or endometritis indicated surgical-site infection.

Febrile morbidity was defined by temperature above 38°C at least 4 hours apart on two or more occasions, excluding the first 24 hours after delivery [29]. Abdominal wound infection was defined by partial or total dehiscence, presence of purulent or serous discharge from the wound with indurations, warmth and tenderness. Endometritis was defined by the presence of fever (38°C or above) in association with one or more of the following: uterine tenderness or foul smelling lochia [29]. A diagnosis of surgical site infection was reached by using criteria put forth by Centers for Disease Control and Prevention (CDC) [30-32].

In both groups, the bladder catheter was removed after 24 hours. The occlusive dressing which was applied in theatre was removed after 48 hours and the wound was left open. Assessment of for signs of infection was done on days 3, 7 and 30 post-operatively. We selected these three days because; on day 3 the patient is normally discharged from the hospital if she is doing well, day 7 is when stitches on the wound are removed and day 30 is for final follow up according to CDC definition of surgical site infection. However, before discharging our patients, we educated them on the signs of infection so that they could come any time they feel ill or notice signs suggestive of infection. Assessment was done by measuring body temperature at axilla and performs physical examination. The diagnosis of surgical site infection was made if the infection occurs within 30 days of follow up. Patients with surgical site infection were managed by using hospital treatment guideline.

#### Secondary outcome measure

There were no secondary outcome measures (such as side effects of drugs) simply because the study did not involve a new drug or a new regimen. It was just a comparison of efficacy of a single dose over repeated doses of the same drug combinations.

### Statistical analysis

All data were extracted from patients’ files and used to fill in a case record form designed for this study. The recorded data included socio-demographic characteristics, obstetric history and labor history if any (stage of labor, state of amniotic membrane-whether intact or ruptured) and information on the HIV status of all women was extracted from either the antenatal cards or the patients’ files. Regarding testing for HIV, the current policy is that every woman admitted at Bugando Medical Centre is requested to test for HIV. A trained counselor was available in the labour room to counsel all women coming for delivery. Testing was done according to Tanzania Ministry of Health recommendation. Other patient information was added during follow-up.

Participants who could not attend their follow up days were traced and assessed. We made contact through their own mobile phone numbers or their husbands’, or nearby relatives or by communicating with a ten cell leader since we had their physical addresses. Study participants were reminded to attend their follow-up days by phone call a day before.

Data were double entered into computer using EpiData version 3.1 (CDC, Atlanta, USA) according to codes given. Data were checked for discrepancies and then exported to STATA version 11 (college Station, Texas, USA) for analysis. Descriptive statistics were used to summarize participants’ characteristics. Cumulative incidence was calculated as the following proportion: number of cases of SSI noted over total number of participants in each study group. Time to develop post-caesarean infection was noted and used to calculate the incidence rate (IR) of post-caesarean infection. IR was calculated by using a number of study subjects with infection divided by total person-days of follow up in each study group. Intention-to-treat analysis was used to determine the equivalence between a single dose and multiple dose regimens; we set the equivalence margin at 5%. The difference in proportion with its corresponding 95% confidence interval was computed. The equivalence was considered statistically significant at two-tailed if the 95% confidence interval of the proportion difference lies within the pre-specified equivalence margin of ± 5%.

## Results

### Characteristics of the study population

**Figure 1:**
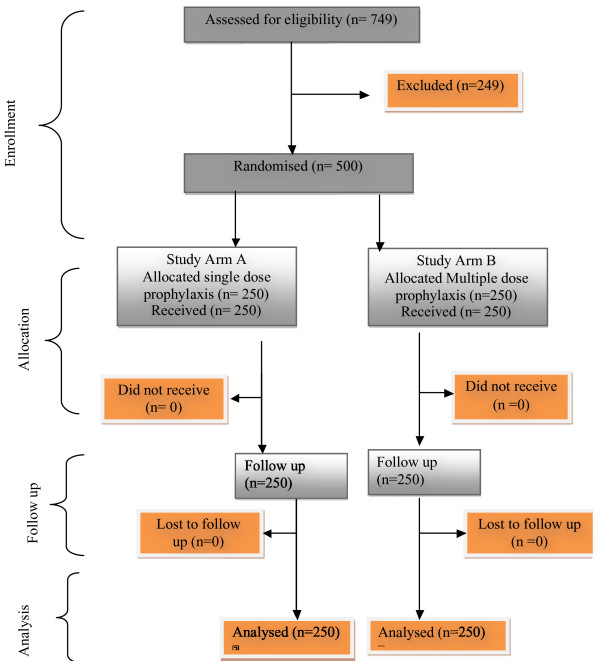
**Participant**’**s flow chart.**

This study was conducted between October 2011 and May 2012. A total of 749 pregnant women were assessed for eligibility. Of these, 249 participants were excluded from the study (220 participants did not meet the inclusion criteria, 21 participants declined to participate in the study and 8 participants were unconscious). Therefore 500 pregnant women were randomly allocated into two arms; 250 received a single dose regime and 250 received multiple dose regimes (Figure 1). No participants in either arm were lost to follow-up. There was an even distribution of the participants with respect to demographic and other baseline characteristics in both arms (p-value >0.05) as shown in Table 1.

**Table 1:** Background parameters in the two groups of women subjected to emergency caesarean section using two different prophylactic regimens

**Variable**	**Single dose (N = 250)**	**Multiple doses (N = 250)**	***p*-value**
**Age of participants**			
20 years and below	38 (15.2%)	42 (16.8%)	0.612
21-30 years	146 (58.4%)	135 (54.0%)	
Above 30 years	66 (26.4%)	73 (29.2%)	
**Gravidity**			
Primegravida	77 (30.8%)	83 (33.2%)	0.565
Multigravida	173 (69.2%)	167 (66.8%)	
**Body mass index (kg/m**^**2**^**)**			
Normal weight	55 (22%)	67 (26.8%)	0.123
Overweight	112 (44.8%)	120 (48.0%)	
Obese	83 (33.2%)	63 (25.2%)	
**Presence of caesarean section scar**			
NO	152 (60.8%)	163 (65.2%)	0.308
YES	98 (39.2%)	87 (34.8%)	
**Amniotic membrane during operation**			
Intact	121 (48.4%)	92 (36.8%)	0.009
Ruptured	129 (51.6%)	158 (63.2%)	
**Duration of operation(minutes)**			
≤60 minutes	151 (60.4%)	140 (56.0%)	0.319
>60 minutes	99 (39.6%)	110 (44.0%)	

### Equivalence of single dose and multiple dose regimens

Post caesarian section surgical-site infection occurred in 12/250 (4.8%) among those that received single dose compared to 16/250 (6.4%) of those that received multiple doses. An absolute proportion difference of 1.6%, with 95% confidence interval (CI) -2.4 – 5.6% was observed. Since 95% CI was not within the pre-specified equivalence margin of ± 5%, we failed to accept the equivalence hypothesis.

**Figure 2:**
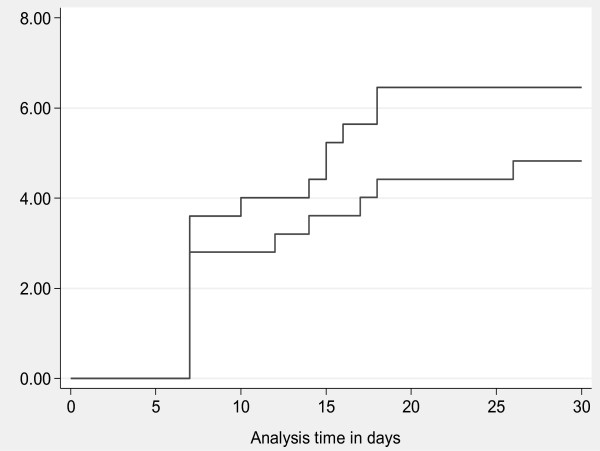
**Kaplan**-**Meier cumulative incidence of Post caesarean infection.**

Participants who used the single dose regime had lower cumulative incidences than those who used multiple doses, (4.8% [95% CI 2.2 – 7.4] versus 6.4% [95% CI 3.4 – 9.4]) as shown in Figure 2.

### Incidence of post-caesarean infection

The incidence rates of post-caesarean infections were 1.7 and 2.3 per 1000 person-days in a single dose and multiple dose regimens respectively with the incidence rate ratio (IRR) of 0.74 [95% CI 0.32 – 1.65 p-value = 0.2146].

## Discussion

This study demonstrated that the administration of intravenous single dose of gentamicin (3 mg/kg) in combination with 500 mg of metronidazole and multiple doses of the same regimen given within 24 hours before emergency caesarean section for prevention of infection are not statistically equivalent. The administration of pre-operative single dose antibiotic prophylaxis has shown to have lower cumulative incidence of surgical-site infection compared to multiple doses. Previous studies have reported that there is no added benefit of using multiples doses over single dose of antibiotics for prophylaxis of SSI [23,33]. For this reason the administration of pre-operative single dose antibiotic prophylaxis for emergency caesarean is recommendable. This is also supported by the 1998 Swedish and Norwegian consensus on the use of antibiotic prophylaxis in surgery, which states that the choice of antibiotics should be generally conservative. Antibiotics used for therapy should be avoided in prophylactic regimens. In most cases, surgical antibiotic prophylaxis should be given as a single dose, and in no case should the prophylaxis time exceed 24 hours [34].

Costs of medications are an important parameter especially is resource-limited settings such as ours where most of the patients do not have health insurance. Multiple doses regime is associated with higher medication cost than single dose regime. Furthermore, previous studies have shown the low prevalence of low-level resistance to gentamicin in our setting [35,36]. Therefore, when considering medication cost and drug-susceptibility pattern of the common bacteria causing surgical-site infection in our setting, single dose regime for prophylaxis is the rational choice.

In addition, the use of single dose regime reduces workload to the nurses, especially at night when few nurses are on duty in the ward. The severe shortage of health care workers is a well-known problem in many resource-limited settings [37]. For instance, the total of staffing in the health sector of Tanzania stands at 35% of the actual need [38]. This causes the few available nurses to be overwhelmed by their duties and sometimes fail to attend to the patients in a timely manner. Consequently, the use of single dose regimen is particularly pertinent for settings with scarcity of health care workers.

## Conclusion

The administration of intravenous single dose of gentamicin (3 mg/kg) in combination with 500 mg of metronidazole and multiple doses of the same regimen given within 24 hours before emergency caesarean section for prevention of infection are not statistically equivalent. Therefore, we recommend the administration of pre-operative single dose antibiotic prophylaxis for emergency caesarean, as this regimen showed to have lower cumulative incidence of surgical-site infection with reduced staff workload and minimized medication cost as compared to multiple doses.

### Ethical approval

This trial did not involve new drugs but only determined the efficacy of a single dose regime versus multiple dose regimes; however GCP and Declaration of Helsinki were observed. Clearance for conducting this study was sought from the Bugando Research Ethics Committee on 22nd August 2011 ref: BREC/001/39/2011.

An informed consent was requested from participants after explaining the study aims. For literate women, the consent information was provided before providing a copy of the consent form that each participant was required to sign.

For non-literate women, the consent information was read in full, and participants were required to thumb print on the consent form to signify their acceptance to participate in the study. A nurse or doctor who was not part of the study was around to witness the verification counseling process for illiterate women.

Participation was voluntary, and those who declined to participate were still entitled to the standard care provided to all women in the labor ward.

## Abbreviations

BMC: Bugando medical centre; CRF: Case record forms; GCP: Good clinical practice; MRSA: Methicillin resistant *Staphylococcus aureus*.

## Competing interests

The authors declare that they have no competing interests. Funding has been received from the Ministry of Health, United Republic of Tanzania, as an unrestricted educational grant for a postgraduate thesis.

## Authors’ contributions

FML provided major contributions in concept, study design, literature review, data collection and entry and drafting of the manuscript. ANM, SEM contributed to study design, writing of the manuscript, and literature searching, and BRK and ETK contributed to concept, study design, data analysis, and writing of the manuscript. All authors read and approved the final manuscript.

## Pre-publication history

The pre-publication history for this paper can be accessed here:

http://www.biomedcentral.com/1471-2393/13/123/prepub

## Prior publication

The protocol for this trial was published in BioMed Central 2012. Lyimo et al.: Efficacy of single dose of gentamicin in combination with metronidazole versus multiple doses for prevention of post-caesarean infection: study protocol for a randomized controlled trial. *Trials* 2012 13:89.
